# Adult nutrient shortage impairs female reproduction via the attenuated juvenile hormone signaling during vitellogenesis in *Helicoverpa armigera* (Lepidoptera: Noctuidae)

**DOI:** 10.1093/jisesa/ieaf094

**Published:** 2025-10-28

**Authors:** Haojie Zheng, Xiangya Liu, Rongbo Huang, Qiaofeng Su, Yingchuan Peng, Haijun Xiao, Wanna Zhang

**Affiliations:** Institute of Entomology, Jiangxi Agricultural University, Nanchang, China; Institute of Entomology, Jiangxi Agricultural University, Nanchang, China; Institute of Entomology, Jiangxi Agricultural University, Nanchang, China; Institute of Entomology, Jiangxi Agricultural University, Nanchang, China; Institute of Entomology, Jiangxi Agricultural University, Nanchang, China; School of Grassland Science, Beijing Forestry University, Beijing, China; Institute of Entomology, Jiangxi Agricultural University, Nanchang, China

**Keywords:** nutrition, reproduction, juvenile hormone, vitellogenesis

## Abstract

In insect reproduction, vitellogenesis is a prerequisite to oocyte maturation and critically depends on nutrient acquisition, supporting reproductive success. In *Helicoverpa armigera* (Hübner 1809), a hallmark of female reproduction is that vitellogenin (Vg) synthesis is initiated following adult eclosion, and juvenile hormone (JH) plays a principal role in this process. The adults commonly visit flowers to feed on nectar, which implies the possible linkage between adult nutrient and vitellogenesis. The preliminary experiment demonstrated that the supplemental nutrition with 10% honey during vitellogenesis significantly shortened the preoviposition period, extended the oviposition duration, and improved female fecundity. Ovary dissection showed that the ovarian development was somewhat delayed in water-fed females, while their ovarian degradation occurred in advance. Consistent with this, on days 1 and 2 of vitellogenesis, the water-fed females possessed a larger proportion of previtellogenic follicles in the ovaries and higher triglyceride content than those in honey-fed individuals. On days 3 and 4, the *Vg* transcription was significantly downregulated in the fat body of water-fed females. Besides, the water feeding during vitellogenesis resulted in an attenuated JH biosynthesis and a notable decline in intrinsic JH content. The expressions of JH pathway genes, *Met* and *Kr-h1*, in the fat body were reinforced in honey-fed females at day 4 of vitellogenesis. These results elucidated the promoting effect of adult nutrient on female reproduction, and we proposed that the adult nutrient shortage impairs female vitellogenesis partly via the attenuated JH signaling.

## Graphical abstract

**Graphical Abstract:**
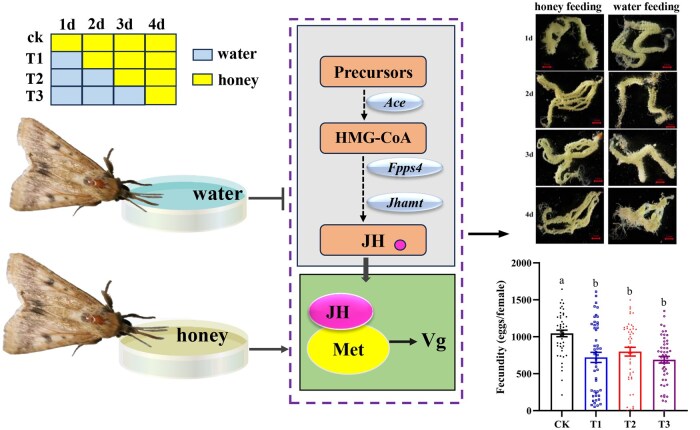

## Introduction

In the wild, newly emerged lepidopteran adults commonly visit flowers to acquire supplementary nutrients from nectar or pollen, although their nutrient intake primarily occurs at the larval stage (He et al. 2022). In many lepidopterans, the supplemental nutrition in the adult stage contributes substantially to reproduction by extending oviposition longevity, increasing female fecundity, and accelerating sexual maturity (Jaumann and Snell-Rood 2019, He et al. 2021, Li et al. 2021, Zhang et al. 2023). In female adults of *Conogethes punctiferalis* (Lepidoptera: Crambidae), supplementary nutrition enhanced fecundity and longevity, with 10% sucrose solution exhibiting the strongest promoting effects; in contrast, females deprived of nutritional supplements did not lay eggs (Zhang et al. 2023). Further studies in *Spodoptera frugiperda* (Lepidoptera: Noctuidae) and *Cnaphalocrocis medinalis* (Lepidoptera: Pyralidae) revealed that supplemental nutrition is beneficial for ovarian development in female moths, leading to higher fecundity (He et al. 2021, Pan et al. 2024). Despite these findings, the intrinsic relationship between female adult diet and ovarian development remains largely unknown.

In female insect reproduction, ovarian maturation requires 2 inextricably linked steps: vitellogenesis and oogenesis, during which vitellogenesis is a prerequisite to oocyte maturation, supporting reproductive success (Wu et al. 2021). Vitellogenesis is characterized by a massive synthesis of yolk protein precursors, mainly vitellogenin (Vg), in the fat body and their uptake by developing oocytes through Vg receptor (VgR)-mediated endocytosis (Raikhel and Dhadialla 1992, Jing et al. 2021). Actually, vitellogenesis occurs in a short period of time and critically depends on nutrient acquisition, during which the female reproductive output is strongly affected by adult nutrition (Smykal and Raikhel 2015, He et al. 2022). This nutritional dependence stems from the high energetic demands of oogenesis, which requires massive recruitment of proteins and lipids for yolk formation (Fruttero et al. 2017). For example, in *Aedes aegypti* (Diptera: Culicidae), only after a blood feeding can the mosquito fat body become competent for massive yolk protein synthesis and secretion (Clifton and Noriega 2012). Altogether, these studies suggested that vitellogenesis in insects is typically a nutrient-limited process, and adult nutrient supplementation is particularly required for rich fecundity in some species.

Accumulated studies have established that insect vitellogenesis is hierarchically regulated by juvenile hormone (JH) and 20-hydroxyecdysone (20E), but their actions vary across insect orders based on reproductive traits (Roy et al. 2018, Swevers 2019, Song and Zhou 2020). In many lepidopterans, including *Manduca sexta* (Lepidoptera: Sphingidae), vitellogenesis is initiated after adult eclosion, and JH plays a dominant role in this process (Telfer 2009). In other lepidopterans such as *Bombyx mori* (Lepidoptera: Bombycidae) and *S. frugiperda*, vitellogenesis is initiated prior to adult ecdysis, wherein 20E appears to play a pivotal role (Swevers and Iatrou 2003, Yan et al. 2025). The molecular action of JH depends on its intracellular receptor, Methoprene-tolerant (Met). JH triggers the dimerization of Met with a transcription factor Taiman (Tai) to constitute an active JH-receptor complex to regulate JH response genes (Charles et al. 2011, Kayukawa et al. 2012, Belles and Santos 2014, Jindra et al. 2015). After the Met/Tai heterodimer binds to the JH response element in the promoter of *Krüppel homolog 1* (*Kr-h1*), Kr-h1 is directly activated and transmits JH signaling to regulate adult reproduction, functioning as a JH early-response gene (Minakuchi et al. 2009, Kayukawa et al. 2012, Belles and Santos 2014). In *Chilo suppressalis* (Lepidoptera: Crambidae), Kr-h1 modulates JH action to promote vitellogenesis and oocyte maturation (Tang et al. 2020). Despite the pivotal role of nutrients in insect vitellogenesis, little is known about how adult nutrient promotes vitellogenesis under hormone regulation.

The cotton bollworm *Helicoverpa armigera* (Hübner 1809) is a destructive polyphagous agricultural pest, with its formidable reproductive capacity being a key factor contributing to its devastating impact (Guo 1998, Riaz et al. 2021). A hallmark of female reproduction in this species is that Vg synthesis is initiated following adult eclosion, and its ovary becomes mature approximately on the third day after eclosion (Zhang et al. 2013, 2014). Their adults usually visit flowers to feed on nectar at dusk (Guo 1998), which implies the possible linkage between adult nutrient and vitellogenesis. Besides, during the vitellogenic period, a high JH titer is coupled with a lack of 20E content in female adults (Zhang et al. 2017, Kang et al. 2019). Further studies in *H. armigera* revealed that knockdown of either *Met* (JH receptor) or *Kr-h1* (early JH-response gene) in female adults suppressed Vg transcription and oocyte maturation, which consolidated the principal role of JH in vitellogenesis (Ma et al. 2018, Zhang et al. 2018).

In this work, the preliminary experiment found that the nutrient shortage during vitellogenesis seriously suppressed Vg transcription and impaired fecundity in female adults, but how nutrient shortage disturbed JH-mediated vitellogenesis remains ambiguous. To address this issue, we determined the effects of nutrient shortage on ovarian development, intrinsic JH titer, and JH signals in female adults during vitellogenesis. The results would provide deep insights into the molecular basis of the crosstalk between adult nutrition and vitellogenesis and accelerate the design of ecological strategies to manage this agricultural pest.

## Materials and Methods

### Insect Rearing

The *H. armigera* used in this study were originally collected from Jiujiang County, Jiangxi Province, China, in 2019. Since then, this strain has been reared on artificial diet and kept in a greenhouse chamber as previously described (Zhang et al. 2021). The rearing conditions were 26 ± 1 °C, 70 ± 10% relative humidity, and a photoperiod of 14:10 (L:D). Larvae were reared in 24-well plates and transferred into 25-mL glass tubes at sixth-instar for pupation (1 larva per tube).

### Nutrition Shortage on Life History Traits during Vitellogenesis

The newly emerged female moths were collected, and each of them was individually paired with a male moth and transferred to a plastic cup (8 cm diameter, 10 cm high). The plastic cups were covered with 1 layer of 10 × 10 cm gauze. A cotton ball saturated with water or 10% of honey solution was placed above the gauze. Since the first day after emergence, the control individuals were continuously fed with 10% honey solution until death, while the treated specimens were fed exclusively with distilled water at the first few days. The feeding durations for different treatment groups were set at 1, 2, and 3 days, respectively, with these groups designated as T1, T2, and T3. After that, the individuals in these treatments were recovered to feed with a 10% honey solution. Then both the gauze and the cotton ball were changed daily to count the number of eggs laid. Besides, the durations of preoviposition, oviposition, and adult longevity were recorded. A total of 45 to 50 female individuals were included in each treatment.

### Nutrient Shortage on Ovary Development during Vitellogenesis

To explore the effect of nutrition shortage on ovarian development during vitellogenesis, the newly emerged female moths were continuously fed with distilled water or a 10% honey solution, separately, from the first day after eclosion. In each treatment, 12 to 15 specimens were randomly selected every day for the daily observation of ovary development, and their ovaries were dissected and observed under an optical microscope (Olympus SZX16). In detail, the numbers of follicles at different developmental stages (previtellogenesis, vitellogenesis, and maturation) were recorded, and the length of the best-developed ovariole in each ovary was measured using a micrometer. Finally, the ratio of ovarian weight (ovary wet weight/whole body weight) was measured to evaluate the ovarian development status.

### Nutrient Shortage on Content of Triglyceride, Protein, and Glycogen

To investigate the effect of nutrient shortage on the intrinsic triglyceride content, the newly emerged female moths were separately fed with distilled water or 10% honey solution. To quantify the triglyceride content in different treatments, specimens were sampled daily from days 1 to 4 in each treatment. Five specimens collected at each time point were pooled to form one replicate, and each treatment was performed 4 times. All samples were frozen immediately in liquid nitrogen and stored at −80 °C until the triglyceride assay. In parallel experiments, samples were prepared with the same method for the determination of protein and glycogen content.

Triglyceride was extracted from the tissue of the abdomen. After weighing, the pooled abdomens from 1 replicate were homogenized in a mixture of n-heptane and isopropanol (v:v = 1:1), and centrifuged at 8,000 × *g* to extract triglyceride. The resultant supernatant was transferred into a new tube. Then the triglyceride content was quantified using a triglyceride content assay kit (Solarbio Life Science, Beijing, China) combined with a colorimetric assay. The absorbance of the sample solution was measured at 420 nm via a Multiskan GO Microplate Spectrophotometer, and the triglyceride content was calculated based on the standard triglyceride. Finally, the content of triglyceride in each sample was calculated as the mass-specific content of triglyceride in the abdomen (mg/g).

The glycogen content was quantified using a Glucogen Content Assay Kit (Solarbio Life Science, Beijing, China) combined with a colorimetric assay. After weighing, the whole insect bodies in 1 replicate were homogenized in lysis buffer containing 30% KOH on ice and then centrifuged to collect the supernatant. The resultant supernatant was harvested and supplemented with 0.12% anthrone reagent. For colorimetric assay, a six-point calibration curve was constructed by serial dilutions of standard glucose solutions (0.1, 0.08, 0.06, 0.04, 0.02, 0 mg/ml). The absorbance of the sample solution was measured at 630 nm, and the glycogen content in each sample was calculated based on the standard curve. The content of glycogen in each sample was calculated as the mass-specific content of glycogen in the whole insect body (mg/g).

The protein content was measured according to a modified Bradford method (Rosendale et al. 2017). In brief, the pooled abdomens from 1 replicate were homogenized in 50 mM Tris-HCl containing 0.1% phenylmethylsulfonyl fluoride on ice and centrifuged to collect the supernatant. The soluble protein was measured with 25 μl of supernatant using Coomassie brilliant blue with bovine serum albumin as the standard. The absorbance of the sample solution was measured at 595 nm. The content of protein in each sample was calculated as the mass-specific protein content in the abdomen (mg/g).

### Nutrient Shortage on JH Signals during Vitellogenesis

To explore the effect of nutrition shortage on JH signals, the newly emerged female moths were fed with distilled water or 10% honey solution, separately, from the first day after eclosion. In each treatment, 10–12 individuals were sampled daily from days 1 to 4, and the tissues of the head, ovary, and fat body were dissected under a stereomicroscope. Specimens collected at each time point were pooled to constitute 1 replicate. All tissues were frozen immediately in liquid nitrogen and stored at −80 °C until RNA isolation. Three independent replicates were included in each treatment.

### RNA Isolation and cDNA Synthesis

Total RNA was extracted with TRIzol Reagent (Thermo Scientific, China). The RNA concentration and quality were checked by Nanodrop 2000 Spectrophotometer (Thermo Scientific, United States) and 1% agarose gel electrophoresis, respectively. After removal of residual genomic DNA, 2 μg of total RNA was used to synthesize the first-strand cDNA with a FastKing RT kit with gDNase (Tiangen, Beijing, China).

### qRT-PCR Analysis

The expression profiles of JH biosynthesis genes (*Ace*, *Fpps4*, and *Jhamt*) and JH signal genes (*Met* and *Kr-h1*) were investigated by qRT-PCR detection. Two house-keeping genes *β-actin* (EU527017) and *Ef-1α* (FJ768770.1) were chosen as the reference genes. The qRT-PCR reaction was carried out using SYBR Green SuperReal PreMix Plus (Tiangen, Beijing, China) through a CFX96 Touch Real-Time PCR detection system (Bio-Rad). In the preliminary experiment, the amplification efficiencies of the target and reference genes were determined using a gradient dilution of the templates. Each reaction for each sample was performed in triplicate and included negative controls without a template. The geometric mean of the Cq values of 2 reference genes was calculated to normalize variations between them (Bustin et al. 2025). The relative expression levels normalized to reference genes were calculated by the comparative 2^−△△CT^ method. The primer pairs for target and reference genes are listed in Supplementary Table S1.

### Nutrient Shortage on JH Titer during Vitellogenesis

To investigate the effect of nutrition shortage on intrinsic JH titer, the newly emerged female moths were separately fed with water or 10% honey solution from the first day after eclosion, and specimens were sampled daily from days 1 to 4 in each treatment. Five specimens collected at each time point were pooled to form 1 replicate, and each treatment was replicated 6 times. JH was extracted following an ultrasonic-assisted n-hexane extraction approach as previously described (Zhang et al. 2017). Next, the intrinsic JH titer (mass-specific content of JH in insect body; ng/mg) was quantified using an Insect JH III ELISA Kit (Zhenke Biological Technology, Shanghai, China) combined with a colorimetric assay. A five-point calibration curve was first constructed by serial dilutions of standard JH III solutions to cover a range from 0.006 to 0.160 ng/ml (Supplementary Fig. S1). Based on the standard curve, the JH content in each sample was determined. At least 5 individuals were contained in each group, and 6 biological replicates were included in each treatment. Additionally, several sample solutions were selected and subjected to serial dilution, and the intrinsic JH contents were determined (Supplementary Fig. S2). Then the dilution curves of standard JH III and sample solutions were compared to verify the reliability of the ELISA technique.

### Statistical Analysis

Statistical analyses were performed using SPSS 18.0 (SPSS, Chicago, IL, United States). Significant differences of life history traits among groups were analyzed using one-way ANOVA followed by Tukey’s honest significant difference test for mean comparison (*P *< 0.05). Moreover, the ovarian weight ratio and the percentage of follicles at different stages were arcsine transformed before ANOVA to meet the assumptions of normality. Two-group datasets were analyzed by Student’s *t*-test, which was applied in the statistical analysis of the content of metabolites, ovarian development parameters, intrinsic JH titer, and target gene expressions.

## Results

### Effects of Nutrition Shortage on Reproduction

**Fig. 1.**
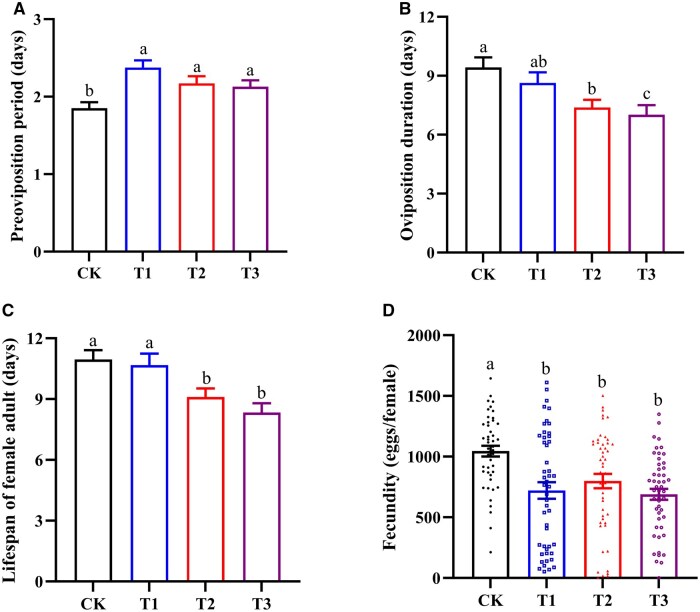
The effects of nutrient shortage on the A) pre-oviposition period, B) oviposition duration, C) lifespan of female adult, and D) female fecundity in *Helicoverpa armigera* female adults. The control individuals are newly emerged female moths continuously fed with 10% honey solution until death. The treated specimens are newly emerged female moths fed exclusively with distilled water at the first few days after emergence. The feeding durations for different treatment groups are set at 1, 2, and 3 days respectively, with these groups designated as T1, T2, and T3. A total of 45 to 50 female individuals were included in each treatment. Significant difference among groups was determined using one-way ANOVA followed by a Tukey’s honest significant difference test for mean comparison. Different letters indicate significant differences among groups (*P *< 0.05).

To explore the effect of nutrition shortage on female reproduction during vitellogenesis, the life history traits of newly emerged females were determined in different treatments. Compared with the honey-fed group, the preoviposition period was significantly extended in newly emerged females continuously starved for 1, 2, and 3 days (*F *= 5.781; df = 3,188; *P *= 0.001) (Fig. 1A), and simultaneously their oviposition durations were shortened by 0.8, 1.2, and 2.4 days, respectively (Fig. 1B) (*F *= 5.079; df = 3,188; *P *= 0.002). Significant differences were also detected in the lifespans of newly emerged females continuously starved for 2 and 3 days, which were decreased by 1.8 and 2.2 days, respectively (Fig. 1C) (*F *= 6.876; df = 3,188; *P *< 0.001). Moreover, when exposed to continuous starvation for 1, 2, and 3 days, the water-fed females exhibited a 31.04%, 23.59%, and 34.05% decline in oviposition, compared to the honey-fed individuals (Fig. 1D) (*F *= 8.117; df = 3,188; *P *< 0.001).

### Effects of Nutrition Shortage on Ovarian Development

**Fig. 2.**
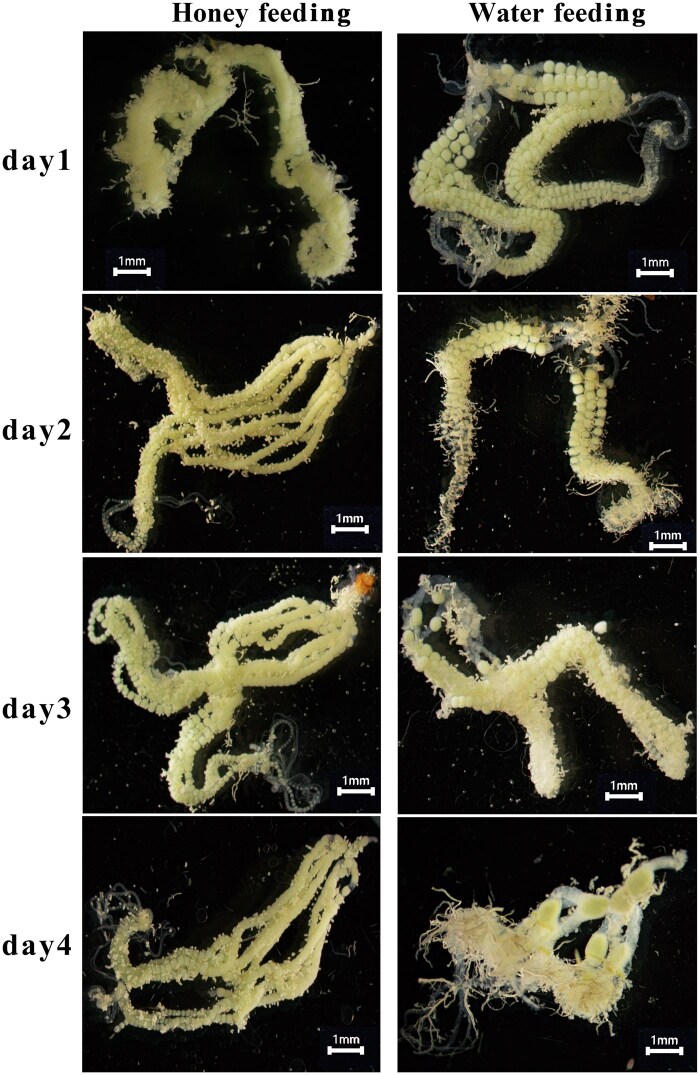
Effects of nutrient shortage on ovarian development during vitellogenesis of *Helicoverpa armigera*. Representative images of ovary morphology after 10% honey feeding, compared with those exclusively fed on water, observed daily from days 1 to 4 post treatment. The scale bar is denoted as 1 mm.

To investigate the effect of nutrition shortage on ovarian development during vitellogenesis, the ovarian morphology was observed daily in different treatment individuals (Fig. 2). The results showed that, at days 1 and 2, the oviducts in honey-fed females were filled with plump oocytes, with almost no gaps between them; while the water-fed females exhibited less-developed oocytes with a distinct contour. On the third day after treatment, the ovary dissection revealed that the ovarian morphology was identical between honey-fed and water-fed individuals, and it was found that the ovaries of both groups became fully mature. A significant difference was observed on day 4; the ovarioles in honey-fed females were still packed with abundant mature follicles, whereas the water-fed individuals showed atrophied ovaries with scarcely any mature follicles in the ovarioles. These morphological differences revealed that the ovarian development was somewhat delayed in water-fed individuals, while their ovarian degradation occurred in advance.

**Fig. 3.**
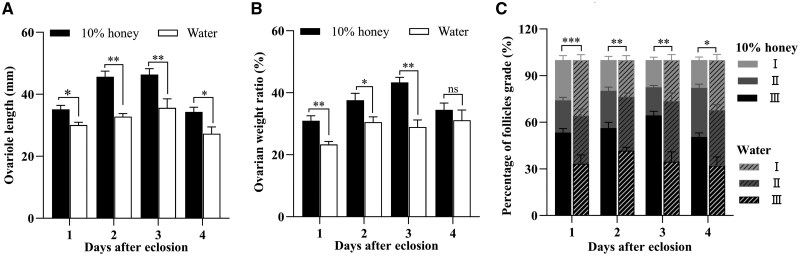
Effects of nutrient shortage on ovarian development during vitellogenesis in *Helicoverpa armigera*. Determination of the length of the best-developed ovariole (A), the ovarian weight ratio (B), and the percentage of follicles at different developmental stages (C) in newly emerged female moths continuously fed on 10% honey solution or water-only from days 1 to 4 post treatment. The developmental status of follicle was divided into 3 grades: (I) previtellogenesis, (II) vitellogenesis, and (III) maturation. Data were presented as mean ± SE, with 12 to 15 specimens in each treatment. Significant differences between treatments at each time point were determined by Student’s *t*-test (*P < 0.05; **P < 0.01; ***P < 0.001; ns, not significant).

The parameters describing the ovarian development status were further determined in different treatment individuals. The results showed that the length of the best-developed ovariole in honey-fed females was remarkably longer than that in water-fed individuals throughout the treatment period (Fig. 3A) (day 1: *t *= 3.685, df = 14, *P *= 0.002; day 2: *t *= 6.286, df = 14, *P *< 0.001; day 3: *t *= 2.294, df = 14, *P *= 0.014; day 4: *t *= 3.224, df = 14, *P *= 0.006). It was notable that the honey-fed females developed the longest ovarioles on the third day after treatment, with a length of 46.40 ± 1.90 mm, significantly longer than those in water-fed individuals (35.60 ± 2.92 mm). The ovarian weight ratio is defined as the proportion of the weight of ovary to that of the entire body, an indicator of potential reproductive capacity. From days 1 to 3 post treatment, the ovarian weight ratio of honey-fed females decreased by 24.70%, 18.74%, and 33.24% respectively, compared with those in water-fed individuals (Fig. 3B) (day 1: *t *= 3.299, df = 11, *P *= 0.007; day 2: *t *= 2.017, df = 13, *P *= 0.045; day 3: *t *= 4.410, df = 11, *P *= 0.001).

Finally, the numbers of follicles at different developmental stages were compared in different treatment individuals (Fig. 3C). The follicles at the previtellogenesis stage (grade I) accounted for a larger proportion in water-fed females at days 1 and 2 than the proportion of those in honey-fed individuals. Moreover, a higher proportion of mature follicles (grade III) was detected in honey-fed individuals relative to the water-fed individuals (day 1: *t *= 5.936, df = 11, *P *< 0.001; day 2: *t *= 4.393, df = 11, *P *= 0.001; day 3: *t *= 7.685, df = 11, *P *< 0.001; day 4: *t *= 2.243, df = 11, *P *= 0.046). These findings revealed that the ovarian development became delayed in females deprived of honey supply.

### Effects of Nutrition Shortage on Content of Triglyceride, Protein and Glycogen

**Fig. 4.**
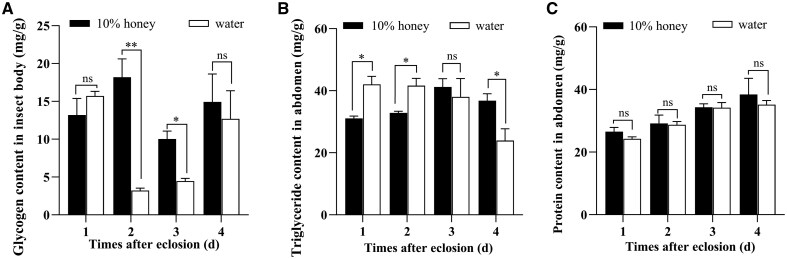
Effects of nutrient shortage on the content of glycogen (A), triglyceride (B), and protein (C) during vitellogenesis. The newly emerged female moths were continuously fed on 10% honey solution or water-only from days 1 to 4. The results were presented as the mean and standard errors of 4 biological replicates, with 5 specimens in each replicate. Significant difference between groups was calculated by Student’s *t*-test (*P < 0.05; **P < 0.01; ns, not significant).

To evaluate the effect of nutrition shortage on nutrient reserve during vitellogenesis, the content of triglyceride, protein, and glycogen was determined in different treatment individuals (Fig. 4). The results showed that the body glycogen content exhibited a drastic fluctuation in water-fed females, while the honey-fed individuals maintained a relatively steady glycogen content (Fig. 4A). A significant difference was detected on days 2 and 3 after treatment, wherein the glycogen content in honey-fed females was 18.20 ± 4.17 and 10.03 ± 1.80 mg/g, respectively, markedly higher than that in water-supplied individuals (3.20 ± 0.54 and 4.47 ± 0.60 mg/g) (day 2: *t *= 6.831, df = 3, *P *= 0.006; day 3: *t *= 5.244, df = 3, *P *= 0.013). It was notable that the triglyceride reserve in honey-fed females was significantly decreased by 26.01% and 21.14%, respectively (day 1: *t* = −3.820, df = 3, *P *= 0.032; day 2: *t* = −3.324, df = 3, *P *= 0.048), in the first 2 days after treatment, compared with the water-supplied individuals. However, this trend exhibited a reversal on days 3 and 4 (Fig. 4B) (day 3: *t *= 0.471, df = 3, *P *= 0.67; day 4: *t *= 3.573, df = 3, *P *= 0.037). Additionally, no significant difference was observed in the intrinsic protein content between the honey-fed and water-fed individuals throughout the treatment period (Fig. 4C).

### Effects of Nutrition Shortage on *Vg* and *VgR* Transcription

**Fig. 5.**
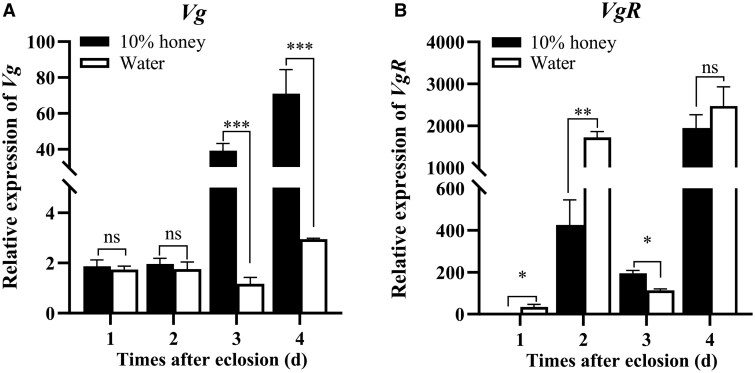
The effects of nutrient shortage on the expression of *Vg* in fat body (A) and *VgR* in ovary (B) during vitellogenesis. The newly emerged female moths were continuously fed on 10% honey solution or water-only from days 1 to 4. The results were presented as the mean and standard errors of 3 biological replicates, with 10 to 12 individuals in each replicate. Significant difference between groups was calculated by Student’s *t*-test (*P < 0.05; **P < 0.01; ***P < 0.001; ns, not significant). Vg, vitellogenin.

Vg is primarily synthesized in the fat body and enters the developing oocytes through VgR-mediated endocytosis (Wu et al. 2021). To explore the effect of nutrition shortage on Vg synthesis and uptake, the transcriptions of *Vg* in fat body and *VgR* in ovary were measured in individuals from different treatments. As shown in Fig. 5, the *Vg* transcription was significantly downregulated by 97.07% and 95.84% in water-fed females on days 3 and 4, relative to the honey-fed group (day 3: *t *= 15.61, df = 2, *P *= 0.004; day 4: *t *= 8.658, df = 2, *P *= 0.013). Besides, honey supply led to a significant reduction of *VgR* expression by 96.51% (*t* = −5.075, df = 2, *P *= 0.037) and 75.47% (*t* = −11.125, df = 2, *P *= 0.008) at days 1 and 2, respectively. However, its expression was induced by 42.04% on day 3 (*t *= 13.651, df = 2, *P *= 0.005).

### Effects of Nutrition Shortage on Intrinsic JH Titer and JH Signals

**Fig. 6.**
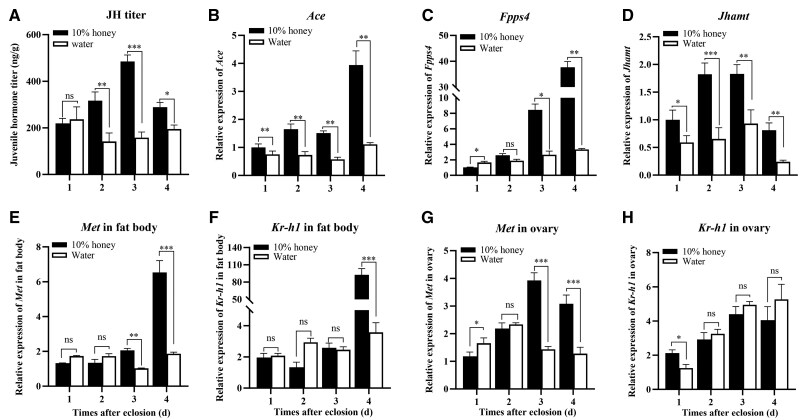
The effects of nutrient shortage on intrinsic JH titer (A), along with the expressions of JH biosynthesis genes (*Ace*, *Fpps4*, and *Jhamt*) in the tissue of head (B to D), and JH signal genes (*Met* and *Kr-h1*) in fat body (E and F) and ovary (G and H). The newly emerged female moths were continuously fed on 10% honey solution or water-only from days 1 to 4. For qRT-PCR analysis, the results were presented as the mean and standard errors of 3 biological replicates, with 10 to 12 individuals in each replicate. For the determination of JH titer, 5 specimens collected at each time point were pooled to form one replicate, and each treatment was replicated 6 times. Significant difference between groups was calculated by Student’s *t*-test (*P < 0.05; **P < 0.01; ***P < 0.001; ns, not significant). JH, juvenile hormone.

To investigate the effect of nutrition shortage on JH content during vitellogenesis, the intrinsic JH titer was first measured in different treatment individuals (Fig. 6A). The honey-fed females exhibited a notably higher JH titer than the water-fed individuals from days 2 to 4, with the JH content increasing by 55.36%, 67.43%, and 32.50%, respectively (day 2: *t *= 4.647, df = 5, *P *= 0.006; day 3: *t *= 11.555, df = 5, *P *< 0.001; day 4: *t *= 3.538, df = 5, *P *= 0.017). Besides, the transcriptions of JH biosynthesis genes, *Ace*, *Fpps4*, and *Jhamt*, were significantly attenuated in water-fed females from days 1 to 4, relative to the honey-fed individuals. For example, on day 1 (ie the first day of vitellogenesis), the JH biosynthesis genes, *Ace* and *Jhamt*, were down-regulated by 7.60% and 24.16%, respectively, when females were subjected to water supply. These results indicated that the JH biosynthesis was remarkably attenuated in water-fed females during vitellogenesis.

Then the effect of nutrition shortage on JH signals during vitellogenesis was investigated. The results showed that the honey supply significantly induced the expression of *Met* on days 3 and 4 in both fat body (day 3: *t *= 15.047, df = 2, *P *= 0.004; day 4: *t *= 10.756, df = 2, *P *= 0.009) and ovary (day 3: *t *= 23.964, df = 2, *P *= 0.002; day 4: *t *= 24.245, df = 2, *P *= 0.002), compared to the corresponding water-fed group (Fig. 6). In honey-fed individuals, the primary JH response gene, *Kr-h1*, was up-regulated by 30-folds in fat body on day 4 (*t *= 15.258, df = 2, *P *= 0.004) and by 41.30% in ovary on day 1 (*t *= 4.698, df = 2, *P *= 0.042). However, no significant difference was observed in *Kr-h1* expression at other time points between treatments.

## Discussion

After eclosion, many lepidopteran adults search for supplemental nutrient resources to promote reproduction, and they mainly collect nectar rich in sugars, vitamins, and amino acids (He et al. 2022). Here, our results in *H. armigera* showed that supplemental nutrition with 10% honey during the vitellogenic period significantly shortened the preoviposition period, extended the oviposition duration, and improved female fecundity. Cumulative studies have established that adult nutrition was beneficial for female reproduction, which has been documented in *C. medinalis* (Pan et al. 2024), *S. frugiperda* (Yang et al. 2025), *C. punctiferalis* (Zhang et al. 2023), *Pseudaletia sequax* (Lepidoptera: Plutellidae) (Marchioro and Foerster 2012), etc. For instance, in *Plutella xylostella* (Lepidoptera: Noctuidae), carbohydrate intake by their female adults increases the oviposition period and fecundity by 6-fold (Marchioro and Foerster 2013). Overall, these findings highlighted the promoting effects of adult supplementary nutrition on multiple reproductive characters in female reproduction. However, the underlying mechanism has not yet been determined.

In insect reproduction, vitellogenesis constitutes a pivotal step of ovarian maturation and involves abundant Vg synthesis in the fat body and Vg uptake by the developing ovary (Raikhel and Dhadialla 1992, Wu et al. 2021, Jing et al. 2021). In *H. armigera*, vitellogenesis is initiated at the adult stage, and its ovary becomes mature approximately on the third day after eclosion (Zhang et al. 2013, 2014). Our results of ovary dissection showed that the ovarian development was delayed in water-fed females, whereas their ovarian degradation occurred in advance, compared with honey-fed individuals. Consistent with this, the water-fed females possessed a larger proportion of previtellogenic follicles in the ovaries. Conversely, in female moths of *C. punctiferali*s and *C. medinalis*, a carbohydrate-rich diet promoted ovary development, leading to a higher fecundity (Pan et al. 2024, Yang et al. 2025). Besides, our results revealed that, on days 3 and 4 of vitellogenesis, the *Vg* transcription was significantly downregulated in the fat body of water-fed females. Actually, vitellogenesis occurs in a short period and critically relies on nutrient supply. During this process, nutrient deficiency has a detrimental impact on female fertility. Likewise, during the oviposition cycle of *Romalea microptera* (Orthoptera: Romaleidae), starvation of females in the early or middle of this cycle reduced Vg production and inhibited oocyte growth (Hong et al. 2005). In *Tribolium castaneum* (Coleoptera: Tenebrionidae), starvation of female beetles resulted in a block in Vg synthesis (Parthasarathy et al. 2010). Considering these documents, we speculated that the shortage of *Vg* supply to ovarian development would ultimately result in the precocious degradation of ovaries in water-fed females of *H. armigera*.

In addition to the pivotal role of adult diet in *H. armigera* vitellogenesis, little is known about how adult supplemental nutrient promotes vitellogenesis under hormone regulation. In this study, the water supply during vitellogenesis led to a notable decline in intrinsic JH content, as well as an attenuated JH biosynthesis. Likewise, in *A. aegypti*, starvation of 4-day-old females caused a significant decrease in JH synthesis than those in sugar-fed females (Perez-Hedo et al. 2014). Whereas a blood meal elevated the circulating JH titer in *Rhodnius prolixus* (Hemiptera: Reduviidae), mainly driven through insulin neuropeptides (Leyria et al. 2022). It has been established that the nutritional signaling pathways, insulin/IGF signaling (IIS) along with target of rapamycin (TOR), are primary nutrient sensors that are responsible for detecting nutrient status and regulating nutrient utilization (Wu and Brown 2006, Baker and Thummel 2007). Further studies demonstrated that IIS and TOR manipulated insect vitellogenesis by means of direct regulation or interaction with the JH signaling cascade (Roy et al. 2018, Zhu et al. 2020, Leyria 2024). In *T. castaneum*, *Periplaneta americana* (Blattodea: Blattidae), and the German cockroach *Blattella germanica* (Blattodea: Blattidae), IIS and TOR signaling promote the transcriptions of *Jhamt* (JH biosynthesis enzyme), *Met*, and *Kr-h1*, further activating *Vg* expression and oocyte maturation (Parthasarathy and Palli 2011, Abrisqueta et al. 2014, Zhu et al. 2020). Besides, our results showed the reduced expressions of JH pathway genes, *Met* and *Kr-h1*, in water-fed females at day 4 of vitellogenesis, which was consistent with the precocious ovary degradation detected at the same time. In *H. armigera*, JH acts as the major gonadotrophic hormone promoting vitellogenesis (Zhang et al. 2017, Ma et al. 2018, Zhang et al. 2018). In water-fed females during vitellogenesis, we speculated that the attenuated JH biosynthesis as well as the reduced JH titer may be attributed to the feedback regulation of IIS and TOR signaling in response to the adult nutrient shortage, which further suppressed Vg production and impaired female fecundity.

These findings suggest that manipulating adult nutrition by reducing nectar-rich flowering plants in agricultural landscapes may be an effective strategy to suppress *H. armigera* populations by suppressing JH-mediated vitellogenesis (Wäckers et al. 2007). Another strategy is a food-based “push-pull” strategy, which uses nectar plants as food attractant to trap the female moths of *H. armigera* (Cook et al. 2007). Additionally, targeting JH-regulated vitellogenesis may also offer novel avenues for biopesticide development, such as JH analogues or nutritional interference agents.

**Fig. 7.**
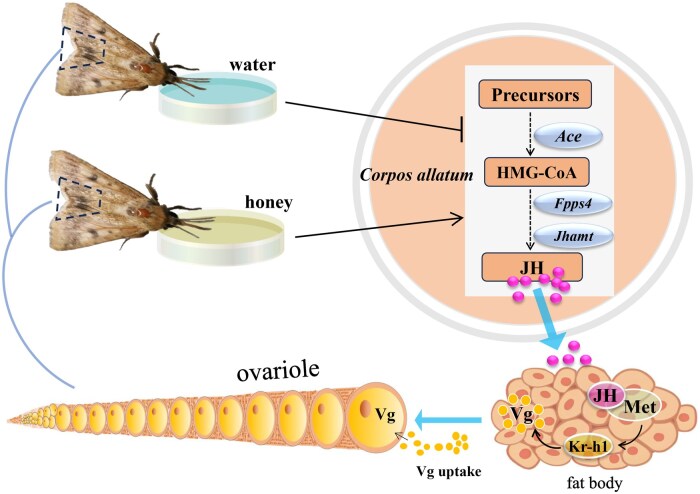
The deduced mechanism of how nutrition shortage suppressed Vg synthesis via the attenuated JH biosynthesis and JH signaling. Met, methoprene-tolerant; Kr-h1, Krüppel homolog 1; Ace, acetoacetyl-CoA thiolase; Fpps, farnesyl diphosphate synthase; Jhamt, juvenile hormone acid methyltransferase; Vg, vitellogenin.

In conclusion, this work made a comprehensive analysis of reproductive characters, ovarian development, and hormone regulation in *H. armigera* female adults subjected to nutrient shortage during vitellogenesis. The results revealed that adult nutrient shortage impaired female vitellogenesis partly via the attenuated JH signaling. Then, we proposed a deduced mechanism of how nutrition shortage suppressed Vg synthesis via the attenuated JH biosynthesis and JH signaling (Fig. 7). Since adult nutrition is particularly required for rich fecundity in *H. armigera* females, a deep insight into this mechanism would contribute to the development of ecological strategies to manage this agricultural pest.

## Supplementary Material

ieaf094_Supplementary_Data
