# Supplementary material for: Adult nutrient shortage impairs female reproduction via the attenuated juvenile hormone signaling during vitellogenesis in Helicoverpa armigera (Lepidoptera: Noctuidae)
Source: J Insect Sci. 2025 Oct 28;25(5):ieaf094. doi: 10.1093/jisesa/ieaf094 (PMC12571479; doi:10.1093/jisesa/ieaf094)
Supplement: ieaf094_Supplementary_Data [file ieaf094_supplementary_data.zip › Table S1.docx]

**Table S1** Primers used for qRT-PCR.

| **Primer name** | **Forward primer (5′-3′)** | **Reverse primer (5′-3′)** | **Product length (bp)** | **Primer efficiency (%)** | **Linear regression** | **Regression coefficient (R^2^)** | **Accesion**  **number** |
| --- | --- | --- | --- | --- | --- | --- | --- |
| *Actin* | GCGACATCAAGGAGAAGCTG | CGTCGCACTTCATGATGGAG | 142 | 104.48 | y = -3.219x+49.136 | 0.9974 | EU527017 |
| *EF-1α* | GGGCAAGGAAAAGATTCACA | GGCCTCCTTCTCGAACTTCT | 125 | 101.43 | y = -3.288x+50.45 | 0.997 | FJ768770.1 |
| *Met* | CTATCCAGTGCAATGCCACC | CCCAGCGGACATCATCTTTG | 187 | 105.86 | y = -3.1889x+22.441 | 0.9977 | KJ825895.1 |
| *Kr-h1* | GTTACCACCGACTCCTCCT | AACGATGATGGGGCAAAGG | 165 | 103.21 | y = -3.2472x+23.522 | 0.9962 | KJ825896.1 |
| *Vg* | CCTTGGTGCCCTACTCTCAA | TGGGTCAAAGCGATGTCTCT | 214 | 103.05 | y = -3.251x+51.822 | 0.9935 | JX504706.1 |
| *VgR* | CCTGCAACAACAGCACATGTT | AAGGGACAGACGCATTGCTT | 140 | 96.49 | y = -3.409x+52.754 | 0.9942 | KC181922.2 |
| *Ace* | TCCAAAAGGAGAATGGAACG | TCCCCATCAGCATATCCAAT | 145 | 99.44 | y = -3.3354x+27.736 | 0.992 | XM_021328343.3 |
| *Fpps4* | GAGACTGGCAAGCACATTGA | CAGCATTTTGTAAGCCAGCA | 123 | 99.35 | y = -3.3376x+23.084 | 0.991 | XM_021326473.3 |
| *Jhamt* | ACGCTCTGCACTGGGTTAAT | CGGTACACGTCAAACACAGG | 121 | 98.75 | y = -3.3522x+23.651 | 0.9985 | AB127945 |

**Figure S1** The calibration curve of JH III. A five-point calibration curve was constructed by serial dilutions of standard JH Ⅲ solutions. The values of X-axis plotted on the X-axis were represented as JH III concentration (ng/L).


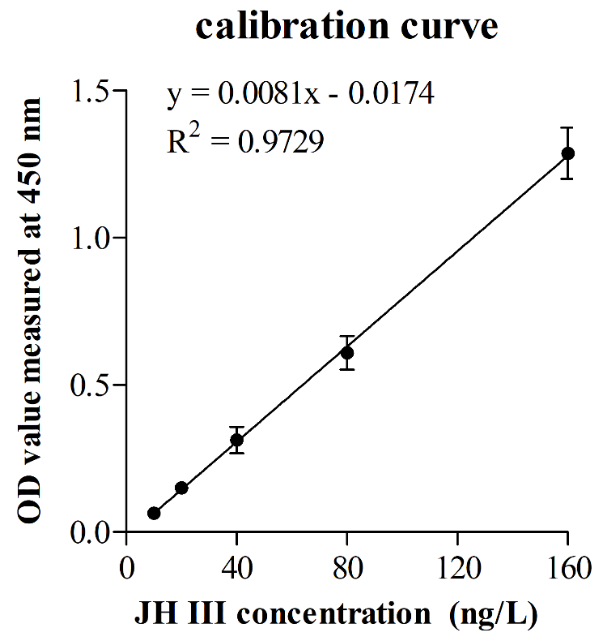


**Figure S2** The curve of gradient dilution of sample solutions. The values plotted on the X-axis were represented as the concentration of gradient dilution of sample solutions. Two sample solutions were selected and serial diluted (1.33-fold, 2-fold, 4-fold dilution) to determine the JH III content.
